# Prevalence and Risk Factors of Preterm Birth Among Pregnant Women Admitted at the Labor Ward of the Komfo Anokye Teaching Hospital, Ghana

**DOI:** 10.3389/fgwh.2022.801092

**Published:** 2022-06-06

**Authors:** Enoch Odame Anto, Wina Ivy Ofori Boadu, Stephen Opoku, Ebenezer Senu, Valentine Christian Kodzo Tsatsu Tamakloe, Augustine Tawiah, Frank Ankobea, Emmanuel Acheampong, Agartha Odame Anto, Michael Appiah, Yaw Amo Wiafe, Max Efui Annani-Akollor, Christian Obirikorang, Otchere Addai-Mensah

**Affiliations:** ^1^Department of Medical Diagnostics, Faculty of Allied Health Sciences, College of Health Sciences, Kwame Nkrumah University of Science and Technology, Kumasi, Ghana; ^2^Centre for Precision Health, School of Medical and Health Sciences, Edith Cowan University, Perth, WA, Australia; ^3^Department of Obstetrics and Gynaecology, Komfo Anokye Teaching Hospital, Kumasi, Ghana; ^4^Department of Molecular Medicine, School of Medicine and Dentistry, College of Health Science, Kwame Nkrumah University of Science and Technology, Kumasi, Ghana; ^5^Department of Obstetrics and Gynecology, Ho Teaching Hospital, Ho, Ghana; ^6^Department of Medical Laboratory Science, Accra Technical University, Accra, Ghana

**Keywords:** preterm birth, labor ward, adverse pregnancy outcomes, risk factors, Komfo Anokye Teaching Hospital, prevalence

## Abstract

Preterm birth is a global epidemic and a leading cause of neonatal mortality in Sub-Saharan Africa. We evaluated the prevalence and risk factors of preterm birth among women attending the labor ward for delivery at a tertiary hospital in Ghana. This comparative cross-sectional study was conducted among a cohort of 209 pregnant women admitted to the labor ward of the Komfo Anokye Teaching Hospital (KATH). Pregnant women who delivered between 28 and 36 completed weeks of gestation were classified as preterm delivery whereas those who delivered after 37–42 completed weeks were described as term. Sociodemographic, clinical, and obstetric data were collected from patient's folder and hospital archives. Categorical variables were analyzed and expressed as frequencies and proportions. We determined the association between obstetric factors and preterm delivery with multiple logistic regressions. Significance level of the strength of association was determined at *p*-value < 0.05. of the 209 participants, the prevalence of preterm birth was 37.3% (78/209) whereas 62.7% (131/209) delivered at Term. Intrauterine growth restriction (IUGR) [*aOR* = 2.15, 95% *CI* = (1.819.55), *p* = 0.0390], HELLP (hemolysis, elevated liver enzymes and low platelet count) syndrome [*aOR* = 3.94, 95% *CI* = (1.64–9.48), *p* = 0.0020], early gestational obesity [aOR = 2.11, 95% *CI* = (1.31–11.92), *p* = 0.0480] and preeclampsia [*aOR* = 4.56, 95% *CI* = (1.63–12.76), *p* = 0.004] were identified as independent risk factors of preterm birth. Prevalence of preterm birth was high among women attending labor admission at the Komfo Anokye Teaching Hospital and this was independently influenced by IUGR, HELLP syndrome, early gestational obesity, and preeclampsia. Identifying early signs of adverse pregnancy outcomes would inform the need for management policy to prevent high prevalence of preterm births.

## Introduction

Preterm birth is a global epidemic and a leading cause of neonatal mortalityas well as a major contributor to long-term adverse health outcomes (1). Preterm birth occurs when a baby is born before the 37th completed week of gestation. Although preterm birth has an unknown cause, its etiologic phenotypes are broadly categorized into spontaneous preterm birth (natural onset of labor or preterm premature rupture of membranes) and provider-initiated preterm birth (induction of labor or pre-labor elective cesarean for maternal or fetal indications) (2, 3). The multiple etiologies such as individual and environmental factors makes preterm birth prediction and prevention a difficult process in antenatal care (1). According to a multi-country study undertaken in low-and-middle income countries (LMICs), spontaneous preterm birth is the most common and babies born preterm have a greater risk of death (4, 5). The greater risk of dying has been mostly associated with neonatal infections (6).

In comparison to term infants, preterm neonates are more susceptible to short and long-term neurocognitive and motor deficits, as well as malnutrition, chronic illnesses, and early death (7). Preterm newborns' chances of survival vary greatly depending on where they are born (6). An African infant's risk of neonatal death owing to preterm delivery difficulties is over 9 times higher than that of a European newborn (2). The global prevalence of preterm birth varies between 5 and 18% among 184 countries (1, 8). Preterm birth affects about 15 million of the 130 million babies born each year around the world. The highest rates of preterm birth occur in Sub-Saharan Africa and Asia, which account for half of the world's births (1). Thus, Sub-Saharan Africa and Asia are responsible for more than 60% of the world's preterm babies and more than 80% of the world's 1.1 million neonatal fatalities per year (1). Preterm birth has been associated with sociodemographic, clinical, and obstetric variables in previous investigations (9, 10). Due to prematurity disproportionate contribution to infant death, it has also been identified as a major impediment to achieving the Millennium Development Goals (MDG)-4 target (11).

Despite an increase in the global incidence of preterm births, data available from developing countries such as Ghana is limited (12). Most countries, particularly those with low-and-middle incomes, have seen a rise in preterm birth rates during the last 20 years, according to the few data available. Identifying and understanding the risk factors for preterm birth has the potential to help address this problem and also aid to achieve the Sustainable Development Goal 3 target of reaching the neonatal mortality rate to 12 per 1,000 live birth by 2030. Currently, there is a paucity of data on preterm births and possible risk factors at the Komfo Anokye Teaching Hospital. This study determined the prevalence of preterm birth and its associated risk factors among pregnant women admitted to the labor ward at the Komfo Anokye Teaching Hospital, Ghana.

## Materials and Methods

### Study Design/Settings

This hospital-based comparative cross-sectional study was conducted at the Komfo Anokye Teaching Hospital (KATH), Kumasi Ghana. KATH is the second major tertiary Hospital positioned in the middle belt of Ghana. The facility has over 1,200 bed capacity and serves as a referral center for other hospitals in the middle and northern belt of Ghana. From KATH records unit, annual total deliveries range from 8,438 to 11,188 for the past 5 years. KATH has 3 delivery wards: the main delivery ward where a majority of deliveries take place; and a delivery ward each in the special ward and the high dependency unit. The average monthly deliveries at KATH is about 900; 80% occurring in the main labor suite, 15% in the special ward and 5% in the high dependency unit.

### Ethical Consideration

Ethics approval was given by the Committee on Human Research, Publications, and Ethics (CHRPE) at the school of Medical Sciences of Kwame Nkrumah University of Science and Technology (CHRPE/SMS/KNUST: CHRPE/AP/205/21). Written permissions were sought from the management of facilities in which data and information were collected, thus sought from the KATH. Written informed consent were obtained from participants and legally authorized representatives of the participants.

### Study Population and Subject Selection

Using a simple randomized sampling technique, a total of 209 singleton pregnant women who are between 18 and 45 years and had reported for delivery at the labor ward between May to June 2021 were selected for this study. The classification of preterm and term was done by a qualified consultant Gynecologist. Pregnant mothers who delivered between 28 and 36 completed weeks of gestation were classified as preterm birth and whereas pregnant mothers who delivered at 37–42 completed weeks were classified as term birth. The gestational age (GA) in the present study was defined in weeks as the duration of pregnancy before birth based on menstrual history (date of the first day of the last menstruation), clinical examination, and ultrasonography (measuring the crown-rump length of the fetus during a first-trimester). Intrauterine growth restriction (IUGR) was defined as the estimated fetal weight less than the 5th centile along with abnormal Doppler velocimetry values (above 95th centile). IUGR was further examined determined by the measurement of the mother's belly from the top of the pubic bone to the top of the uterus (fundal height). Prenatal Doppler ultrasound was also done consultant sonographer. Prior to delivery, structured, and close-ended questionnaires were used to obtain information on sociodemographic data such as age, level of education, ethnicity, occupation, and economic income. Patients' folders and the hospitals' database were used to obtain all the other information needed concerning the study participants clinical and obstetric history. Pregnant women who did not give informed consent, those who were medically unstable and those with the twin pregnancies were excluded.

### Sample Size Calculation

The sample size was obtained by the formula:

n = $\frac{Z^{2}\;p\left(1-p\right)}{e^{2}}$, Where: Z is the standard normal variate at a confidence interval of 95% = 1.96.

p is the prevalence of preterm birth, which was 9.0% in a study by Agbeno et al. (13), at the Cape Coast Teaching Hospital, e is the margin of error = 0.05.

n (Minimum number of participants) = $\frac{1.96^{2}\;\left(0.09\right)\left(1-0.09\right)}{0.05^{2}}$ = 126.

Hence, a minimum of 126 participants were needed for the study.

A 95% confidence level, 50% response distribution, and 5% margin of error is employed in the calculation of the sample size. To increase statistical power, a total of 209 participants were included in the study.

### Statistical Analysis

The collected data obtained were entered, coded, edited, and cleaned in Microsoft Excel 2016. All statistical analyses were performed using the Statistical Package for Social Sciences (SPSS) Version 26.0 (Chicago IL, USA) and GraphPad Prism version 5.0 (GraphPad Software, San Diego California USA, www.graphpad.com). A simple bar chart was used to illustrate the prevalence of preterm birth among study participants. Chi-square test/Fischer's Exact test and the binary logistic regression analysis were employed to test for associations and the strength thereof between the dependent variable (preterm birth) and independent variables. The *p-*values < 0.05 were considered statistically significant for all analyses.

## Results

**Figure 1:**
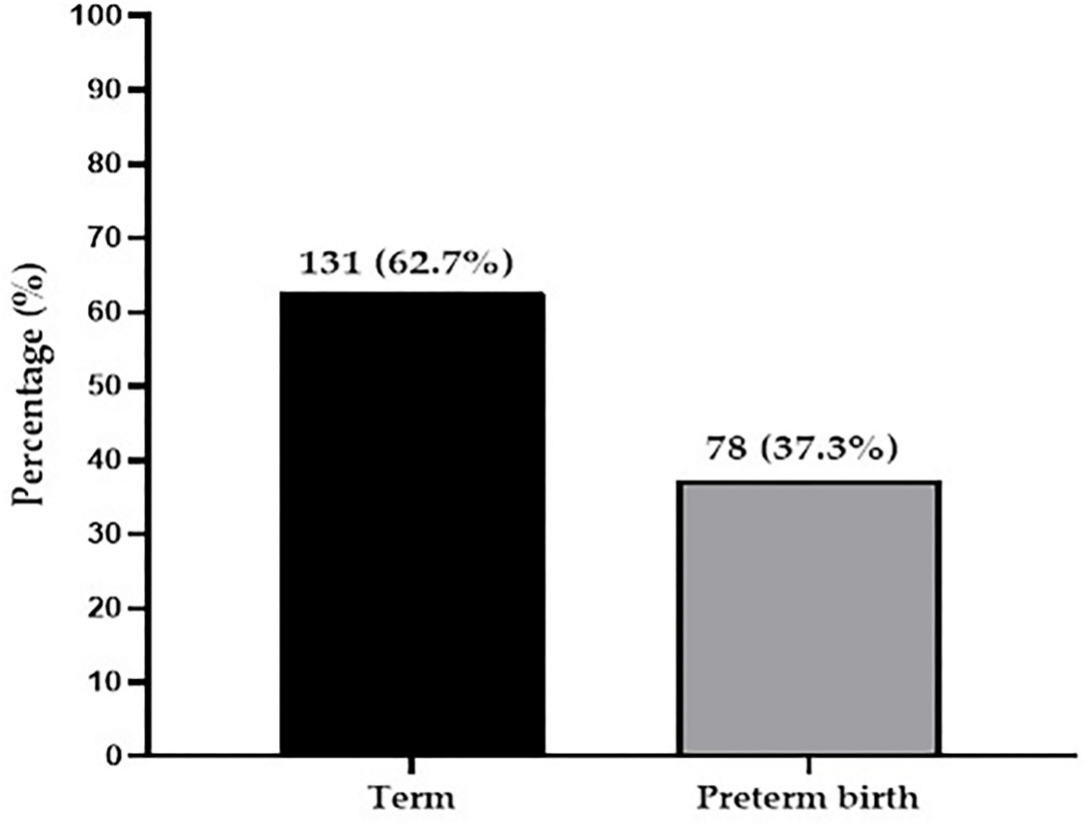
Prevalence of preterm birth among study participants.

Of the 209 participants, the prevalence of preterm birth was 37.3% whereas 62.7% delivered term babies (Figure 1).

Table 1 shows association of preterm birth with sociodemographic and obstetric factors. Occupational status of women was significantly associated with preterm birth (*p* = 0.0350). However, this study did not find any significant association between pregnant women's maternal age (*p* = 0.1950), parity (*p* = 0.7540), gravidity (*p* = 0.2060), their level of education (*p* = 0.1820), marital status (*p* = 0.8620), ethnic group belonging to (*p* = 0.1700), their economic income (*p* = 0.2140), and preterm birth.

**Table 1:** Association of pretermbirth with sociodemographic and obstetric factors among pregnant women.

**Variable**	**Total (*n* = 209)**	**Preterm (*n* = 78)**	**Term (*n* = 131)**	***p*-value**
**Maternal age category**				0.1950
18 to 25	57 (27.3)	17 (21.8)	40 (30.5)	
26 to 33	86 (41.1)	38 (48.7)	48 (36.6)	
34 to 44	66 (31.6)	23 (29.5)	43 (32.8)	
**Parity**				0.7540
Nulliparous	77 (36.8)	31 (39.7)	46 (35.1)	
Primiparous	48 (23)	18 (23.1)	30 (22.9)	
Multiparous	84 (40.2)	29 (37.2)	55 (42.0)	
**Gravidity**				0.2060
Primigravida	59 (28.2)	26 (33.3)	33 (25.2)	
Secundigravida	150 (71.8)	52 (66.7)	98 (74.8)	
**Level of education**				0.1820
None	23 (11.0)	10 (12.8)	13 (9.9)	
Junior high school	99 (47.4)	35 (44.9)	64 (48.9)	
Senior high school	59 (28.2)	18 (23.1)	41 (31.3)	
Tertiary	28 (13.4)	15 (19.2)	13 (9.9)	
**Marital status**				0.8620
Married	178 (85.2)	66 (84.6)	112 (85.5)	
Unmarried	31 (14.8)	12 (15.4)	19 (14.5)	
**Ethnic group**				0.1700
Akan	162 (77.5)	60 (76.9)	102 (77.9)	
Mole Dag	38 (18.2)	17 (21.8)	21 (16.0)	
Ga Adangbe/Ewe	9 (4.3)	1 (1.3)	8 (6.1)	
**Occupation status**				**0.0350**
Unemployed	24 (11.5)	8 (10.3)	16 (12.2)	
Informal	154 (73.7)	52 (66.7)	102 (77.9)	
Formal	31 (14.8)	18 (23.1)	13 (9.9)	
**Economic income (GHC)**				0.2140
<500.00	147 (70.3)	50 (64.1)	97 (74.0)	
500–1000	28 (13.4)	15 (19.2)	13 (9.9)	
>1000.00	8 (3.8)	4 (5.1)	4 (3.1)	

*p-value of < 0.05 was considered statistically significant. P-values were computed using the Chi square/ Fischer's Exact test where appropriate. The bold values indicate p-values which are statistically significant*.

Table 2 shows association of preterm with clinical and perinatal factors. There was a significant association between mode of delivery of women (*p* **< ** 0.0001), intrauterine growth restriction (IUGR) (*p* **< ** 0.0001) HELLP syndrome (Hemolysis, elevated liver enzymes, and low platelet count) (*p* **< ** 0.0001), early gestational BMI (*p* = 0.0050), and preeclampsia (*p* **< ** 0.0001) and preterm birth. On the contrary, no significant association was found between family history of hypertension (*p* = 0.2503), previous history of hypertension (*p* = 0.2890), history of spontaneous abortion (*p* = 0.2340), previous caesarian section (*p* = 0.5680), antenatal visits (*p* = 0.290), and preterm birth.

**Table 2:** Association of preterm birth with clinical and perinatal factors among pregnant women.

**Variable**	**Total (*n* = 209)**	**Preterm (*n* = 78)**	**Term (*n* = 131)**	***p*-value**
**Family History of HTN**				0.2503
No	201 (96.2)	73 (93.6)	128 (97.7)	
Yes	8 (3.8)	5 (6.4)	3 (2.3)	
**Previous history of HTN**				0.2890
No	204 (97.6)	75 (96.2)	129 (98.5)	
Yes	5 (2.4)	3 (3.8)	2 (1.5)	
**History of Spont. Abortion**				0.2340
No	134 (64.1)	54 (69.2)	80 (61.1)	
Yes	75 (35.9)	24 (30.8)	51 (38.9)	
**Previous Caesarian section**				0.5680
No	170 (81.3)	65 (83.3)	105 (80.2)	
Yes	39 (18.7)	13 (16.7)	26 (19.8)	
**Antenatal visits**				0.2901
Sometimes	3 (1.4)	2 (2.6)	1 (0.8)	
Often	206 (98.6)	76 (97.4)	130 (99.2)	
**Mode of delivery**				**<0.0001**
EL C/S	3 (1.4)	2 (2.6)	1 (0.8)	
EM C/S	79 (37.8)	13 (16.7)	66 (50.4)	
SVD	127 (60.8)	63 (80.8)	64 (48.9)	
**IUGR**				**<0.0001**
Yes	148 (75.1)	34 (43.6)	114 (87.0)	
No	61 (24.9)	44 (56.4)	17 (13.0)	
**HELLP syndrome**				**<0.0001**
Yes	129 (75.1)	28 (35.9)	101 (77.1)	
No	80 (24.9)	50 (64.1)	30 (22.9)	
**Early gestation BMI**				**0.0050**
18.5 to 24.9	16 (7.7)	9 (11.7)	7 (5.3)	
25 to 29.9	50 (24.0)	26 (33.8)	24 (18.3)	
30 and above	142 (68.3)	42 (54.5)	100 (76.3)	
**Preeclampsia status**				**<0.0001**
Yes	157 (75.1)	44 (56.4)	113 (86.3)	
No	52 (24.9)	34 (43.6)	18 (13.7)	

*HTN, Hypertension; EL C/S, Elective cesarean section; EM C/S, emergency cesarean section; AVD, Assisted virginal delivery; SVD, spontaneous vagina delivery. IUGR, intrauterine growth restriction; HELLP syndrome, haemolysis elevated liver enzymes and low platelet count. p value < 0.05 was considered statistically significant. P-values were computed using the Chi square/ Fischer's Exact test where appropriate. The bold values indicate p-values which are statistically significant*.

Table 3 depicts sociodemographic predictors of preterm birth. Univariate logistic regression indicated that participants who had completed senior high school education and those who had informal occupation were at increased risk of preterm birth. However, these factors were not independent risk factors after adjusting for possible confounders on multivariate logistic regression analysis (*p* > 0.05).

**Table 3:** Multivariate Logistic regression of sociodemographic and obstetric predictors of preterm birth among study participants.

**Variable**	**cOR**	***p*-value**	**aOR**	***p*-value**
**Level of education**
None	1.50 (0.49–4.55)	0.474	0.34 (0.03–3.40)	0.326
Junior high school	2.11 (0.90–4.93)	0.085	0.32 (0.04–2.55)	0.286
Senior high school	2.63 (1.04–6.63)	**0.041**	0.85 (0.11–5.99)	0.853
Tertiary	1.00	-	1.00	-
**Marital status**
Married	1.00	-	-	-
Unmarried	0.93 (0.43–2.04)	0.862	-	-
**Ethnic group**
Akan	1.00	-	-	-
Mole Dag	0.73 (0.36–1.49)	0.381	-	-
Ga Adangbe/Ewe	4.71 (0.57–38.55)	0.149	-	-
**Occupation status**
Unemployed	2.77 (0.91–8.39)	0.072	1.16 (0.14–9.61)	0.888
Informal	2.72 (1.24–5.97)	**0.013**	2.25 (0.32–15.51)	0.412
Formal	1.00	-	1.00	-
**Economic income**
None	1.88 (0.38–9.39)	0.437	-	-
<500.00	1.94 (0.46–8.08)	0.363	-	-
500–1000	0.87 (0.18–4.17)	0.858	-	-
>1000.00	1.00	-	-	-

*HTP, Hypertension; aOR, Adjusted Odd ratio; cOR, Crude Odd ratio; CI, confidence interval. Binary logistic regression analysis performed to obtain odd ratios. p value of < 0.05 was considered statistically significant. The bold values indicate p-values which are statistically significant*.

Table 4 depicts clinical and perinatal predictors of preterm birth. After adjusting for possible confounders in the multivariate logistic regression analysis, IUGR [*aOR* = 2.15, 95% *CI* = (1.81–9.55), *p* = 0.0390], early gestational obesity [*aOR* = 2.11, 95% *CI* = (1.31–11.92), *p* = 0.0480], HELLP syndrome [*aOR* = 3.94, 95% *CI* = (1.64–9.48), *p* = 0.0020] and preeclampsia [*aOR* = 4.56, 95% *CI* = (1.63–12.76), *p* = 0.004] were identified as independent risk factors of preterm birth.

**Table 4:** Multivariate Logistic regression of clinical and perinatal predictors of preterm birth among study participants.

**Variable**	**cOR (95%CI)**	***p*-value**	**aOR (95%*CI*)**	***p*-value**
**Family History of HTN**				
No	1.00		-	-
Yes	0.38 (0.06–2.37)	0.305	-	-
**Previous HTN**				
No	0.38 (0.06–2.37)	0.305	-	-
Yes	1.00		-	-
**History of Abortion**				
No	1.00		-	-
Yes	1.43 (0.79–2.60)	0.235	-	-
**Previous caesarian section**				
No	1.00		-	-
Yes	1.23 (0.59–2.58)	0.5690	-	-
**Antenatal visit**				
Sometimes	3.42 (0.31–38.36)	0.3190	-	-
Often	1.00		-	-
**Maternal age category (years)**				
18 to 25	1.00		-	-
26 to 33	0.54 (0.26–1.09)	0.0860	-	-
34 to 44	0.79 (0.37–1.70)	0.5530	-	-
**Parity**				
Nulliparous	1.00		-	-
Primiparous	1.12 (0.54–2.36)	0.7590	-	-
Multiparous	1.27 (0.67–2.42)	0.4520	-	-
**Gravidity**				
Primigravida	1.00		-	-
Secundigravida	1.48 (0.80–2.74)	0.2070	-	-
**IUGR**				
Yes	3.57 (1.96–6.51)	**<0.0001**	2.15 (1.81–9.55)	**0.0390**
No	1.00		1.00	
**HELLP syndrome**				
No	1.00		1.00	
Yes	7.17 (3.64–14.11)	**<0.0001**	3.94 (1.64–9.48)	**0.0020**
**Early gestation BMI**				
Normal	1.00		1.00	
Overweight	1.19 (0.38–3.69)	0.7670	0.44 (0.09–2.09)	0.3080
Obese	3.06 (1.07–8.76)	**0.0370**	2.11 (1.31–11.92)	**0.0480**
**Preeclampsia**				
Yes	4.85 (2.49–9.47)	**<0.0001**	4.56 (1.63–12.76)	**0.0040**
No	1.00		1.00	

*Binary logistic regression analysis performed to obtain odd ratios. IUGR, intrauterine growth restriction; HELLP syndrome, haemolysis elevated liver enzymes and low platelet count. HTN, hypertension; aOR, Adjusted Odd ratio; cOR, Crude Odd ratio; CI, confidence interval. p value of < 0.05 was considered statistically significant. The bold values indicate p-values which are statistically significant*.

## Discussion

Preterm birth is a global epidemic and a leading cause of neonatal mortality as well as a major contributor to long-term adverse health outcomes. Although there have been recent interventions to reduce preterm birth and its related complication in developing countries (1, 14), there is a paucity of published data on preterm births at the Komfo Anokye Teaching Hospital. This study, therefore, determined the prevalence of preterm birth and associated factors at the second largest teaching and referral hospital in Ghana.

In the present study, we found a 37.3% prevalence of preterm birth among study participants. The prevalence of 37.3% observed in this study is however higher compared to 18.9 and 18.3% observed by Adu-Bonsaffoh et al. (15) and Wagura et al. (10) in a Ghanaian and Kenyan population, respectively. The difference in prevalence between the present finding and that of previous studies may be attributed to the smaller sample size employed in the present study as compared to a larger sample size used by Adu-Bonsaffoh et al. (15) and Wagura et al. (10). Moreover, could be explained by the distinct approaches in the estimation of the gestational age of the babies. Our study recruited participants who were admitted to the labor ward for delivery while other studies took retrospective data. Contrary to this study findings, Agbeno et al. (13), reported 9.0% prevalence of preterm birth in the Cape Coast Teaching Hospital in Ghana. Although the prevalence of Agbeno et al. is much lower compared to the current study, it was observed that the prevalence of preterm birth reported by Agbeno et al. was as low as 0.5% in 2012 but increased sharply to 6.0% in 2017 and 9.0% in 2019. This supports the evidence that the prevalence of preterm birth continue to rise (1), and therefore might have accounted for the higher prevalence in the current study.

Intrauterine growth restriction (IUGR) is a frequent complication in preterm infants and is the cause of most elective late-preterm deliveries (16). In IUGR, the fetus is under nourished for gestational age and this increases the frequency of premature babies who are delivered at preterm (17). The present study observed that participants who had IUGR were 2-fold increased odds of preterm birth. The finding is consistent with a cross-sectional study conducted in Ethiopia by Tesfa et al. (18). The mechanism, which underpins this finding could be attributed to uteroplacental under perfusion that might have resulted in growth restriction of the fetus (18).

In this study, we found early gestational obesity as an independent risk factor for preterm birth. Pre-pregnancy obesity is known to be associated with the risk of delivering preterm babies (19). A cross-sectional study by Parker et al. (20) found obesity as a risk factors for preterm birth, which is consistent with the current study finding. However, the exact physiological mechanism of the effect of early gestational obesity on preterm obesity is still unclear. The current understanding could be that the change in the myometrium from a quiescent to a contractile state is accompanied by a shift from the activation of adipokines and cytokines (21, 22). Maternal obesity creates a unique *in utero* environment, which could impair the placental transcriptome and have potentially adverse consequences for placental structure and function, which might also contribute to the mechanism of preterm birth (23).

The pregnancy complications are known to increase the risk of poor pregnancy outcomes for both mother and baby. In this study, pregnant women who had preeclampsia were ~5 times increased odds of delivering preterm babies. The finding of preeclampsia among pregnant women being associated with preterm birth is consistent with a cross-sectional study by Davies et al. (24), who reported a significant association between preeclampsia and preterm birth. Another cross-sectional study by Koike et al. (25) found that the recurrent risk of preterm birth was due to preeclampsia. Preeclampsia, which is characterized by high blood pressure and proteinuria, is detrimental to both mother and the growing fetus resulting in future cardiovascular risk. The study finding suggests that maintaining optimal control of preeclampsia should be regarded as an important component of antenatal care. This could be addressed through a combination of effective screening, advice, and education, together with facilitating risk factor reduction and the use of antihypertensive medication.

Women with HELLP syndrome have been reported to have higher rates of preterm birth (26). In the present study, the risk of preterm birth associated with HELLP syndrome may be due to its association with preeclampsia (26). Although HELLP syndrome is sometimes considered to be a form of severe preeclampsia, the major distinct features include abnormal liver function, moderate-to-severe thrombocytopenia accompanied by microangiopathic haemolytic anemia, disrupted or destroyed erythrocytes on peripheral smear, and symptoms such as epigastric pain, nausea, and vomiting (27). Our study found that pregnant women with HELLP syndrome were approximately 4-fold increased odds of preterm birth. This finding is consistent with findings from previous retrospective cross-sectional study by Lisonkova et al. (27).

Despite the interesting findings observed in the present study, there were some limitations. Due to the smaller sample size employed in this study, the prevalence of preterm delivery might be overestimated. In addition, recruiting participants from a referral teaching hospital might have contributed to the overestimation of preterm deliveries. Moreover, the cross-sectional study design used limits our study to generalize this finding. A larger sample size and a prospective cohort study designareneeded to understand the casual-effect relationship between preterm birth and the possible risk factors.

## Conclusion

The prevalence of preterm births is high among pregnant women admitted to the labor ward of the Komfo Anokye Teaching Hospital. This was independently influenced by maternal obesity and adverse pregnancy complications such as intrauterine growth restriction, preeclampsia, and HELLP syndrome. Early identification of these adverse pregnancy outcomes may prompt management strategies to mitigate the risk of premature infants from preterm deliveries.

## Author's Note

Preterm birth is a global epidemic and a leading cause of neonatal mortality as well as a major contributor to long-term adverse health outcomes. Preterm birth occurs when a baby is born before the 37th completed week of gestation. While the majority of preterm births are caused by unexplained preterm labor or spontaneous preterm pre-labor rupture of the amniotic membranes, the causes are attributed to multiple etiologies. According to a multi-country study undertaken in low-and-middle income countries, babies born preterm have a greater risk of stillbirth. This study focuses on evaluating sociodemographic, clinical and obstetrics characteristics of pregnant women admitted to the labor ward for delivery. Self-structured questionnaire was employed to obtain sociodemographic data including age, marital status, economic income and occupation. We accessed the patient's folders and medical records for addition information. We found high prevalence of preterm births in mothers admitted at the labor ward. After adjusting for possible cofounders in multivariate logistic regression model, we identified obesity and adverse maternal and perinatal complication contribute to preterm births. We make an assumption that adverse pregnancy complications if identified early would create opportunity for preventive approach and management of mothers who will be at risk of preterm births.

## Data Availability Statement

The raw data supporting the conclusions of this article will be made available by the authors, without undue reservation.

## Ethics Statement

The studies involving human participants were reviewed and approved by the Committee on Human Research, Publications and Ethics (CHRPE) at the school of Medical Sciences of Kwame Nkrumah University of Science and Technology. The patients/participants provided their written informed consent to participate in this study.

## Author Contributions

EOA, WIO, and OA-M conceptualized and designed the study and wrote the manuscript. EOA, WIO, AT, and FA performed the research data collection and laboratory analysis. EOA, SO, and ES performed the data analysis and interpretation. All authors reviewed the manuscript, read, and approved the final manuscript.

## Conflict of Interest

The authors declare that the research was conducted in the absence of any commercial or financial relationships that could be construed as a potential conflict of interest.

## Publisher's Note

All claims expressed in this article are solely those of the authors and do not necessarily represent those of their affiliated organizations, or those of the publisher, the editors and the reviewers. Any product that may be evaluated in this article, or claim that may be made by its manufacturer, is not guaranteed or endorsed by the publisher.
